# Immunohistochemical Evaluation of FGD3 Expression: A New Strong Prognostic Factor in Invasive Breast Cancer

**DOI:** 10.3390/cancers13153824

**Published:** 2021-07-29

**Authors:** Tommaso Susini, Giulia Saccardin, Irene Renda, Milo Giani, Enrico Tartarotti, Jacopo Nori, Ermanno Vanzi, Elisa Pasqualini, Simonetta Bianchi

**Affiliations:** 1 Breast Unit, Gynecology Section, Department of Health Sciences, University of Florence, 50134 Florence, Italy; giulia.saccardin@stud.unifi.it (G.S.); irene.renda@unifi.it (I.R.); milo.giani@unifi.it (M.G.); enrico.tartarotti@unifi.it (E.T.); 2 Diagnostic Senology Unit, Azienda Ospedaliero-Universitaria Careggi, 50134 Florence, Italy; jakopo@tin.it (J.N.); vanzie@aou-careggi.toscana.it (E.V.); 3 Pathology Unit, Department of Health Sciences, University of Florence, 50134 Florence, Italy; elisa.pasqualini@unifi.it (E.P.); simonetta.bianchi@unifi.it (S.B.)

**Keywords:** breast cancer, FGD3 gene, prognostic factors, precision medicine

## Abstract

**Simple Summary:**

Breast cancer is the most incident malignancy and the leading cause of oncological death among women. The recent advances in the treatment of this disease are due to the increasing ability to individualize therapy, in the so called “precision medicine” era. This approach is based on the knowledge of molecular and genetic features of the tumor. Therefore, there is a continuous search for new prognostic factors that may allow us to better stratify patients according to their individual risk. The most promising one seems to be the FGD3 gene expression, which has been shown to be prognostic in breast cancer: the aim of our study was to analyze the prognostic value of FGD3 expression by immunohistochemistry and to compare it with traditional factors. Immunohistochemistry is easy and cheap; to provide by its use a prognostic factor stronger than classical ones may greatly aid in the practical management of this disease.

**Abstract:**

Among new prognostic factors for breast cancer, the most promising one seems to be FGD3 (*Facio-Genital Dysplasia 3*) gene, whose expression improves outcome by inhibiting cell migration. The aim of the study was to evaluate the prognostic role of FGD3 in invasive breast cancer in a series of 401 women, treated at our unit, by evaluating the expression of this gene by immunohistochemistry. Patients with high FGD3 expression showed a significantly better disease-free survival (DFS) (*p* < 0.001) and overall survival (OS) (*p* < 0.001). The prognostic value of FGD3 expression was stronger than that of classical pathologic parameters such as histological grade of differentiation, Ki-67 index and molecular subtype. By multivariate Cox analysis, FGD3 expression was confirmed as significant and independent prognostic factor, ranking second after age at diagnosis (≤40 years) for DFS (*p* = 0.003) and the second strongest predictor of OS, after AJCC Stage (*p* < 0.001). Our data suggest that inclusion of FGD3 evaluation in the routine workup of breast cancer patients may result in a more accurate stratification of the individual risk. The possibility to assess FGD3 expression by a simple and cheap technique such as immunohistochemistry may enhance the spread of its use in the clinical practice.

## 1. Introduction

Breast cancer is the most frequent cancer among women, and its incidence shows an increasing trend, while its mortality is slightly decreasing, although it is still the highest of all cancers in women. The progresses of the recent years in the diagnostic and screening techniques have led to early identification of breast tumors, the continuous renewal of surgical techniques has led, on the other hand, to an increase of conservative treatments, with effectiveness equivalent to the most radical techniques, but with a significant improvement in the social and psychological impact of the operation on the patient. The advances in the number and efficacy of pharmacological protocols as well as the introduction of targeted biological treatments have led to a significant improvement in the prognosis of breast cancer. All these features resulted in a progressive reduction in mortality in recent years with a parallel increase in disease free survival (DFS) and overall survival (OS) [1]. 

The prognostic evaluation of patients with breast cancer is based on a set of widely validated factors such as tumor stage, lymph nodes status, histological grade of differentiation and molecular type. However, it is not uncommon to observe that patients with similar prognostic features display different clinical outcomes. For this reason, research is constantly ongoing to discover new prognostic factors that may allow us to better identify patients at higher risk for recurrence and death from disease.

Among new prognostic factors, the most promising one seems to be the expression of Facio-Genital Dysplasia 3 gene (FGD3), localized on the long arm of chromosome 9 (Chr9q22.31): it has been identified for the first time in 2008 by Hayakawa et al. as a guanine nucleotide exchange factor that targets cell division control protein 42 (CDC42), inducing its activation and modifying cell morphology through the formation of lamellipodia. FGD3 seems to play an inhibiting role on cell migration in neoplastic cells as well as normal ones. Therefore, lower FGD3 expression seems to be correlated with a major risk of cell migration, whereas higher expression seems to be correlated with a minor risk [2]. The role of FGD3 was first described by Cheng et al.: in this study, it was highlighted that FGD3 was the leading protective gene for breast cancer and that the adjacent SUSD3 gene was the second ranked protective gene in the study; furthermore, it was assessed that by silencing one of the two genes, the other was also susceptible to silencing [3]. FGD3 role was also analyzed in a study by Margolin et al. realized to identify and analyze genes that could have a prognostic role in breast cancer: this purpose has been achieved through the analysis of a data set of 1981 cancer samples (METABRIC) [4]. Another study by Ou Yang et al. in 2014 evaluated the use of a new prognostic test (BCAM test) that could be used in all tumor stages and subtypes, consisting of the analysis of expression of FGD3-SUSD3 metagene, other attractor metagenes (CIN, MES, LYM, END, CD68, DNAJB9 and CXCL12), tumor size and the number of positive lymph nodes. Low expressions of FGD3-SUSD3 metagene were found to be associated with poor outcome, even more than low ESR1 expression levels do [5]. In 2017 Willis et al. analyzed a large cohort of invasive breast cancer patients and compared FGD3 expression with other genes that are used as prognostic factors thanks to their association with cell proliferation like MKI67 [6], PCNA [7] and AURKA [8], regardless of molecular subtype and of ER status. Lower expression of FGD3 was associated with a higher rate of lymph node involvement and with decreased DFS and OS. In addition, Willis et al. also demonstrated the prognostic value of FGD3 in head and neck squamous cell carcinoma, lung adenocarcinoma, cervical squamous cell carcinoma, bladder urothelial carcinoma and sarcoma [9]. A previous study by our group described the role of FGD3 gene as a significant and independent prognostic factor in young women with breast cancer. In our previous study, FGD3 expression was evaluated by immunohistochemistry (IHC) and we highlighted the potential clinical benefits of the introduction of such a simple and inexpensive analysis in the daily practice [10]. The aims of the current study were to extend the analysis of FGD3 gene expression by IHC to a series of breast cancer patients at all age treated at our institution and to compare the effectiveness of FGD3 expression to that of traditional prognostic factors. 

## 2. Results

### 2.1. Descriptive Characteristics 

**Figure 1:**
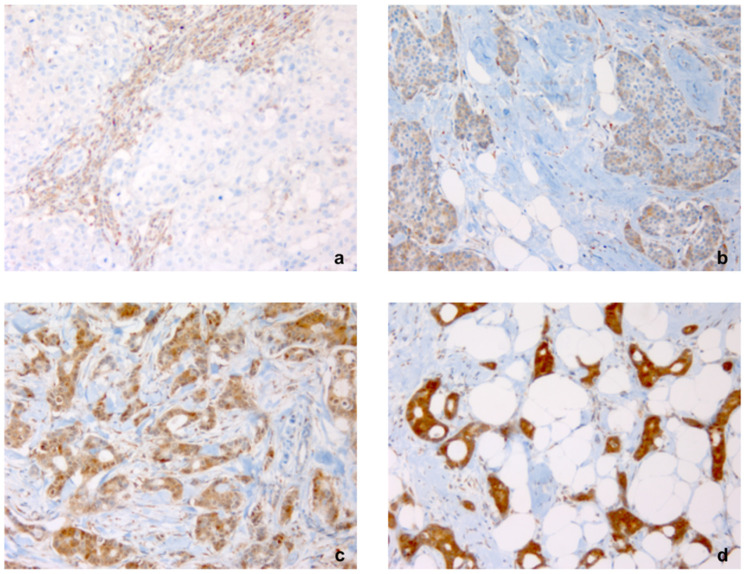
Intensity of immunostaining for FGD3 in breast cancer tissue blocks. Note: Staining was (**a**) negative (−) with positive control in inflammatory cells, (**b**) low (+), (**c**) moderate (++), (**d**) intense (+++) (×200).

For this study we analyzed 401 patients with invasive breast cancer, with an average age of 52.2 years (range 22–89 years). FGD3 expression was evaluated using the direct immunohistochemistry technique (IHC) (Figure 1).

Distribution of clinical and pathologic features according to FGD3 expression showed significant differences for many parameters, except for use of neoadjuvant chemotherapy and adjuvant radiotherapy. Descriptive data of our population are summarized in Table 1.

During the study period, 60 patients (15.0%) experienced recurrence and 30 patients (7.5%) died for the disease.

### 2.2. Association between Traditional Prognostic Factors and Outcome

We analyzed the association between the most important factors traditionally used for prognostic evaluation in breast cancer and outcome using Cox proportional hazards regression analysis. AJCC Stage (*p* < 0.001 on both DFS and OS), lymph node status (*p* = 0.01 on DFS and *p* < 0.001 on OS), hormonal receptors status (*p* < 0.001 on DFS and *p* = 0.015 on OS), Ki67 expression (*p* = 0.048 on DFS and *p* = 0.008 on OS), histological grade of differentiation (*p* = 0.031 on OS), age at diagnosis (*p* < 0.001 on DFS and *p* = 0.018 on OS) confirmed to be significant prognostic factors (data not shown).

### 2.3. Association between FGD3 Expression and Outcome

Concerning FGD3 expression, 140 (35%) of 401 samples analyzed showed a low FGD3 expression and 261 (65%) showed a high FGD3 expression. 

**Figure 2:**
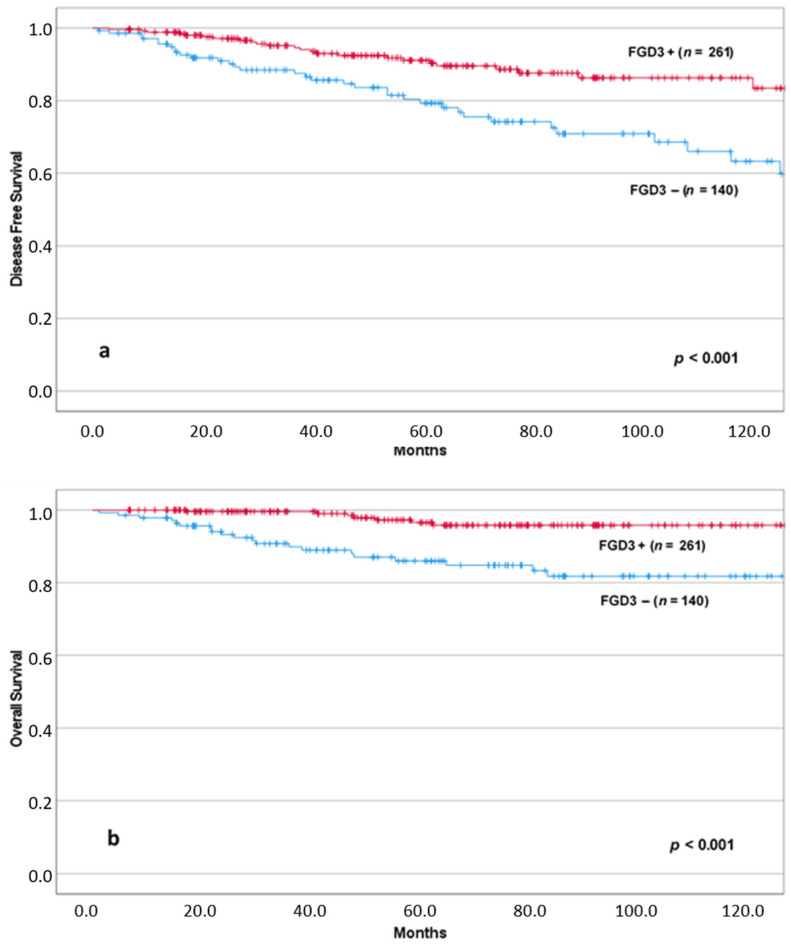
DFS (**a**) and OS (**b**) according to FGD3 expression.

Using the Kaplan–Meier method, we found that patients with low levels of FGD3 expression had significantly reduced DFS (*p* < 0.001) and OS (*p* < 0.001) (Figure 2). 

Low levels of FGD3 expression were associated with reduced DFS e OS either in patients initially treated by surgery as well as in those receiving neoadjuvant chemotherapy. The correspondent Kaplan–Meier estimates are shown in the Supplementary Material (Figures S1 and S2). 

### 2.4. Multivariate Analysis

In Table 2 are shown the results of multivariate Cox proportional hazards regression analysis in which AJCC stage (I–II vs. III–IV), FGD3 expression, hormonal receptors status, age at diagnosis, molecular classification (Luminal A, Luminal B, HER2+, Triple Negative) and histological grade were tested to assess the risk ratio for recurrence and for death from disease and the independence of each variable. Regarding DFS, the young age at diagnosis emerged as the strongest independent indicator of risk of recurrence (*p* < 0.001), followed by the expression of FGD3 (*p* = 0.003), preceding the AJCC stage (*p* = 0. 012). In terms of OS, AJCC stage was ranked first as independent predictor of negative outcome (*p* < 0.001) followed by FGD3 expression (*p* = 0.007). Whereas histological grade and molecular type were not independent predictors of survival by multivariate analysis.

Taking into consideration that age and FGD3 expression were the stronger independent predictors of recurrence, while AJCC stage and FGD3 were the stronger independent predictors of overall survival by multivariate analysis, we further evaluated Kaplan–Meier survival curves, stratifying according these factors.

### 2.5. Association between FGD3 Expression and Outcome Stratified by Age at Diagnosis

**Figure 3:**
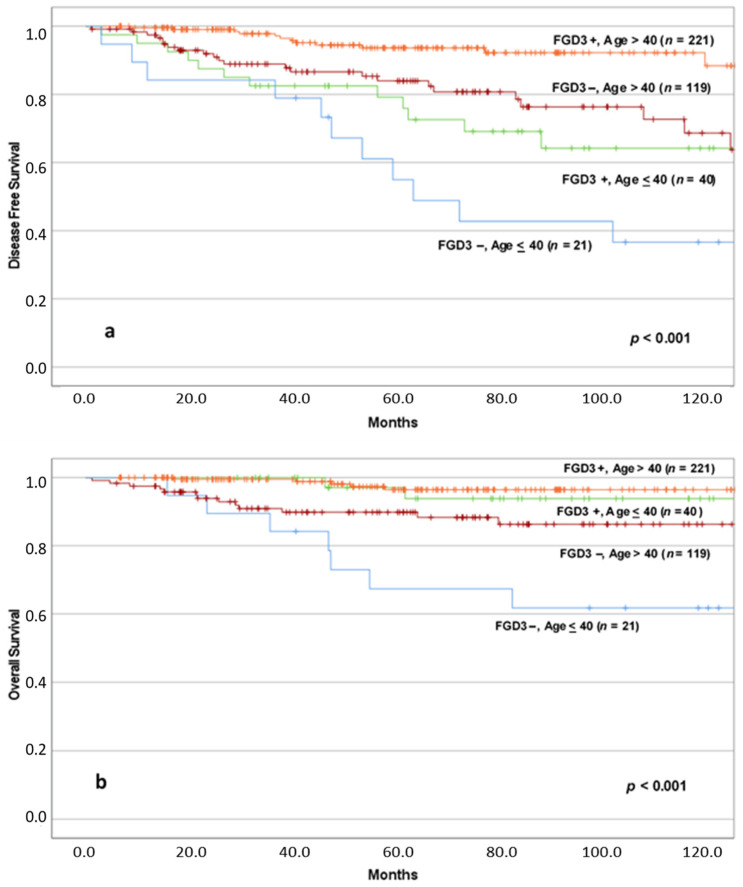
DFS (**a**) and OS (**b**) according to FGD3 expression in age at diagnosis stratified groups. Abbreviations: FGD3+: high FGD3 expression; FGD3−: low FGD3 expression; Age ≤ 40: age at diagnosis ≤ 40 years old; Age > 40: age at diagnosis > 40 years old. Notes: (**a**) FGD3−, Age > 40 vs. FGD3+, Age ≤ 40 (*p* = 0.391); (**b**) FGD3+, Age > 40 vs. FGD3+, Age ≤ 40 (*p* = 0.334), FGD3−, Age > 40 vs. FGD3+, Age ≤ 40 (*p* = 0.290), FGD3−, Age > 40 vs. FGD3−, Age ≤ 40 (*p* = 0.016).

We analyzed the combined impact of FGD3 expression and age on the outcome by Kaplan–Meyer method; survival estimates are shown in Figure 3. 

Concerning DFS, patients diagnosed with cancer over 40 years of age and high FGD3 expression (*n* = 221) had fewer relapses than patients of the same age but with low FGD3 expression (*n* = 119). Similarly, for patients under 40 years of age at diagnosis, there was a lower incidence of recurrence in the high FGD3 expression group (*n* = 40) compared to patients with low FGD3 expression (*n* = 21). Overall, the differences between the four groups in terms of disease-free survival were significant (*p* < 0.001). However, between the group of patients older than 40 years of age with low FGD3 expression and the group of patients younger than 40 years of age but with high FGD3 expression, there was no statistically significant difference (*p* = 0.391).

In terms of OS, patients with high FGD3 expression had fewer deaths, regardless of the age at diagnosis, compared to patients with low FGD3 expression. We can therefore notice the nearly overlapping of the two curves representing patients with high FGD3 expression tumors, with no significant difference in survival (*p* = 0.334). However, it is interesting to underline that between the group of patients older than 40 years of age with low FGD3 expression (*n* = 119) and the group of patients younger than 40 years of age but with high FGD3 expression (*n* = 40), although there was no statistically significant difference (*p* = 0.290), the trend of the curves seems to suggest that the clinical outcome of younger patients with high FGD3 expression is better than that of patients older than 40 years of age at diagnosis but with low FGD3 expression.

### 2.6. Association between FGD3 Expression and Outcome Stratified by AJCC Stage

**Figure 4:**
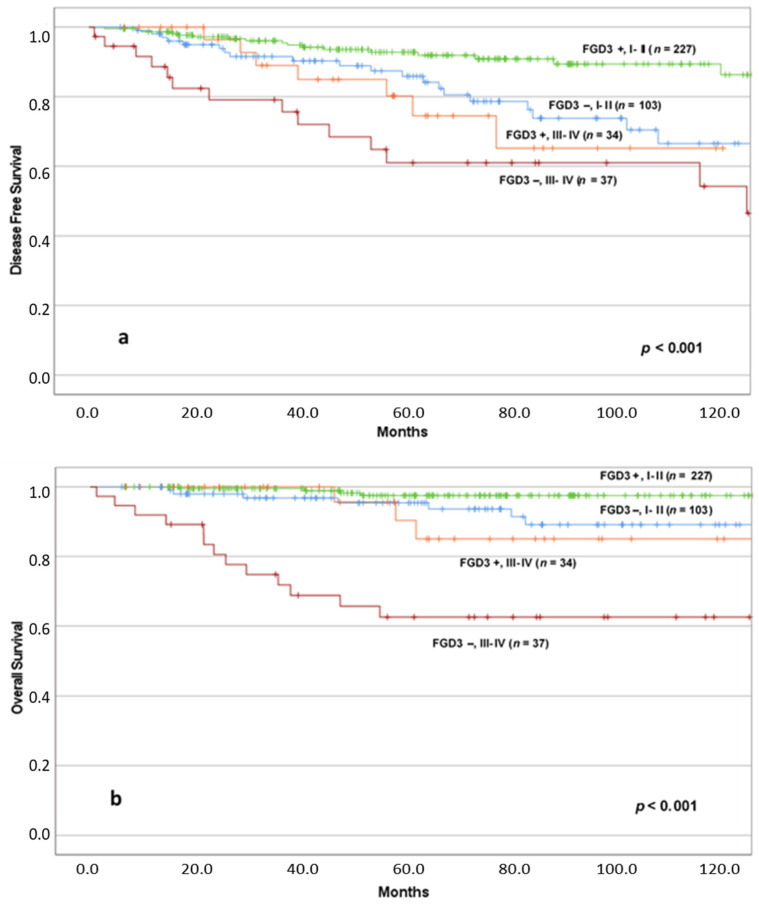
DFS (**a**) and OS (**b**) according to FGD3 expression in AJCC stage stratified groups. Abbreviations: FGD3+: high FGD3 expression; FGD3−: low FGD3 expression; I–II: I, II AJCC Stages; III, IV: III, IV AJCC Stages. Notes: (**a**) FGD3−, I II vs. FGD3+, III IV (*p* = 0.551); (**b**) FGD3−, I II vs. FGD3+, III IV (*p* = 0.342), FGD3−, I II vs. FGD3− III IV (*p* < 0.001), FGD3+, I II vs. FGD3+, III IV (*p* = 0.002).

Then, we stratified our population by AJCC stage, identifying two groups of patients: the one with low AJCC stage (I–II) and the one with high AJCC stage (III–IV), and we analyzed the impact of FGD3 expression on the outcome in the two groups by using Kaplan–Meier method, as shown in Figure 4.

Concerning disease-free survival, patients with early-stage cancer and high FGD3 expression (*n* = 227) had fewer recurrences than patients with tumors at the same stage but with low FGD3 expression (*n* = 103). Similarly, for patients with advanced stage tumors, there was a lower incidence of recurrence in the group with high FGD3 expression (*n* = 34) compared to patients with low expression (*n* = 37). The differences between the four groups in terms of DFS were significant (*p* < 0.001). However, it is noteworthy that between the group of patients with early-stage tumors and low FGD3 expression and the group of patients with advanced stage tumors but with high FGD3 expression, there was no significant difference (*p* = 0.551).

In terms of OS, it is shown that patients with high FGD3 expression and early-stage tumors (*n* = 227) had fewer deaths than both patients with low FGD3 expression and early-stage tumors (*n* = 103) and patients with advanced ones and high FGD3 expression (*n* = 34). The patients who demonstrated the worst clinical outcome were those with advanced tumors and low expression of FGD3 (*n* = 37). The differences between the four groups in terms of overall survival were significant (*p* < 0.001), even if it is possible to appreciate the substantial overlap of the three curves representing patients with high expression FGD3 tumors, with early and advanced stage and low expression of FGD3 and early stage tumors, especially during the first 60 months of follow-up, while after 60 months they demonstrate different trends, better for patients with early stage tumors than those in advanced stage. Again, the comparison between the curves that represent patients with early-stage tumors and low expression of FGD3 and patients with advanced stage tumors and high expression of FGD3 did not reach the statistical significance (*p* = 0.342). Conversely, both for patients with low FGD3 expression (*p* < 0.001) and patients with high FGD3 expression (*p* = 0.002), there was a statistically significant difference between early and advanced stage patients.

### 2.7. Association between FGD3 Expression and Outcome Stratified by Factors Associated with Uncertain Need for Chemotherapy (Node-Negative vs. Node-Positive; Luminal A; Luminal B)

**Figure 5:**
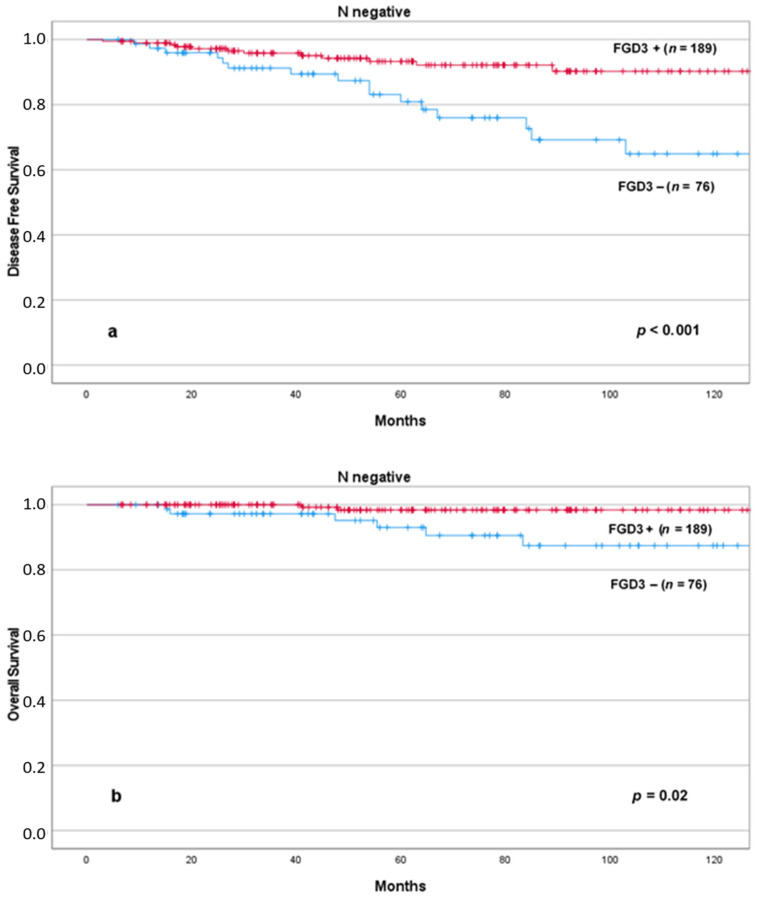
DFS (**a**) and OS (**b**) according to FGD3 expression in node negative patients. Abbreviations: FGD3+: high FGD3 expression; FGD3−: low FGD3 expression.

**Figure 6:**
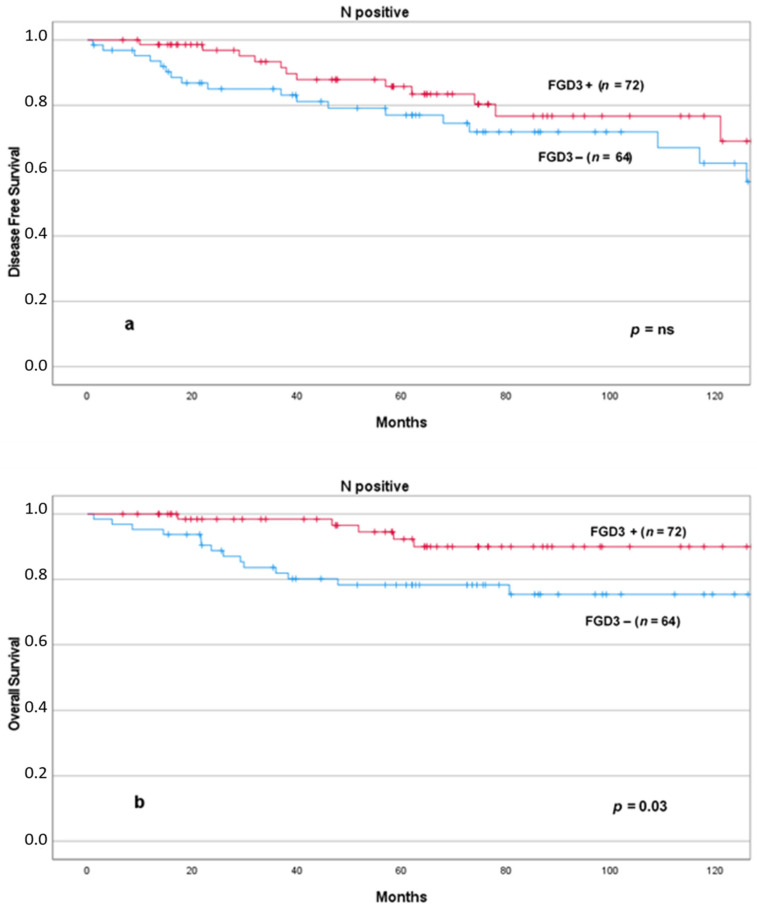
DFS (**a**) and OS (**b**) according to FGD3 expression in node positive patients. Abbreviations: FGD3+: high FGD3 expression; FGD3−: low FGD3 expression.

We then evaluated Kaplan–Meier plots in specific subgroups of patients in which additional prognostic information could be useful to the oncologists to decide whether or not to add chemotherapy. We found that low levels of FGD3 expression were significantly associated with reduced DFS and OS in node-negative patients (Figure 5) and OS in node-positive patients (Figure 6). The combined effect of lymph node status and FGD3 expression in a four lines plot for DFS and OS is also presented in the Supplementary Materials, including pairwise comparison of each curve (Figure S3).

**Figure 7:**
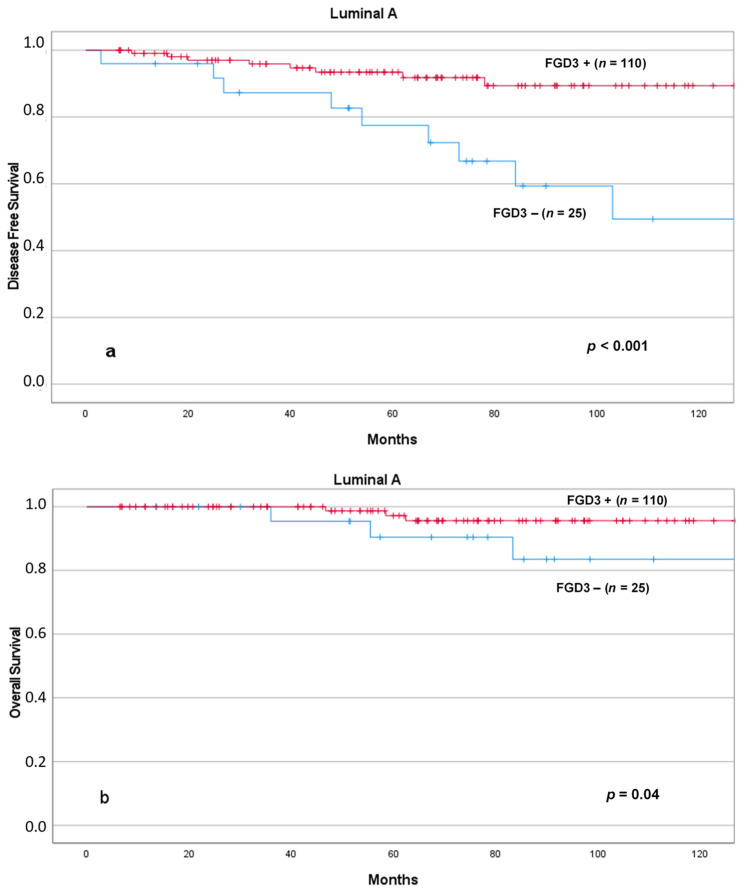
DFS (**a**) and OS (**b**) according to FGD3 expression in patients with Luminal A tumors. Abbreviations: FGD3+: high FGD3 expression; FGD3−: low FGD3 expression.

**Figure 8:**
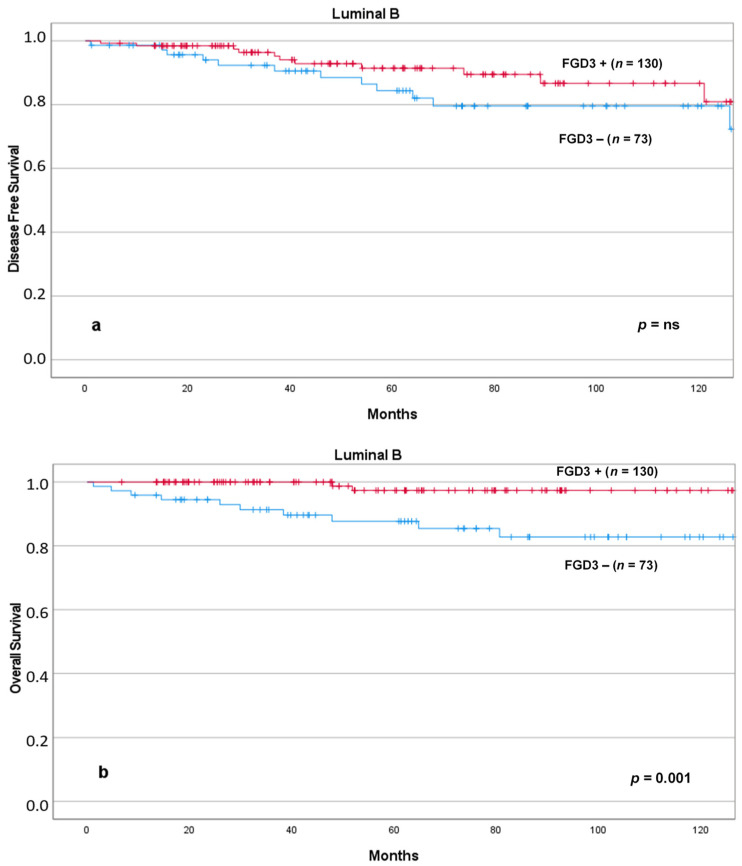
DFS (**a**) and OS (**b**) according to FGD3 expression in patients with Luminal B tumors. Abbreviations: FGD3+: high FGD3 expression; FGD3−: low FGD3 expression.

Similarly, low FGD3 expression was associated with significantly reduced DFS and OS in patients with Luminal A tumors (Figure 7) and OS in patients with Luminal B tumors (Figure 8).

Kaplan–Meier estimates of DFS and OS according to FGD3 expression in triple negative breast cancer patients are shown in Supplementary Materials (Figure S4).

### 2.8. Association between FGD3 Expression and Lymph Node Involvement

We investigated the correlation between FGD3 expression and lymph node involvement by using the Pearson Chi-square test (Table 3). We observed that patients with low levels of FGD3 expression had a higher incidence of lymph node metastases than those with higher levels of FGD3 expression (45.7% versus 27.6%), and the difference was significant (*p* < 0.001). However, there was no statistically significant difference in the extent of lymph node spread (the value of 10 lymph nodes affected by metastases was considered as cut-off) between patients with low and high FGD3 expression. Finally, we analyzed the distribution of the intensity of staining (from − to +++) in patients with negative lymph nodes (*n* = 265) and positive lymph nodes (*n* = 136) by using a Chi-square test: the distribution was significantly different (*p* < 0.001). Similarly, we analyzed the distribution of intensity of staining among patients with positive lymph nodes, dividing the population between patients with involvement of less than 10 lymph nodes and those with involvement of 10 lymph nodes or more: between these two groups there was no statistically significant difference (*p* = 0.327) (data not shown).

## 3. Discussion

The current series of breast cancer patients showed a distribution in terms of clinical and morpho-pathological characteristics that is in line with data from the literature, including the evidence of a more aggressive behavior in young patients [11,12,13]. With regard to the surgical treatment, conservative surgery was performed in 70.1% of cases, whereas 29.9% of the patients underwent mastectomy, in agreement with studies involving larger numbers of patients [12]. With regard to the intervention on axillary lymph nodes, our sample showed a higher rate of sentinel lymph node biopsies (66.8%) compared to total axillary dissection (33.2%), in accordance with data from larger studies that show a significant decrease in total axillary dissections compared to the past [13]. With regard to clinical outcomes, traditional prognostic factors confirmed their role in our study. In fact, significance was achieved by AJCC Stage [14], histological grade [15,16,17], hormonal receptor expression [18,19], proliferation rate (Ki-67 index) [20], age at diagnosis [21,22,23,24], lymph node status [14] on both DFS and OS, in accordance with the literature.

The most relevant finding of our study was the strong prognostic role of FGD3 expression as evaluated by IHC on both DFS and OS, in accordance with the few previous studies [3,4,5,9] that pointed out its value in breast cancer patients. One could question the clinical value of using a single prognostic biomarker, in comparison with existing online resources, such as The Human Protein Atlas, that includes multiple markers [25]. However, it has to be considered that the current research represents the evolution of previous studies based on exploratory analysis of 20,464 possible single-gene biomarkers as categorical variables split on the mean in the METABRIC discovery cohort. These studies identified FGD3 mRNA expression as the highest ranked prognostic gene based on the *p* value for overall survival (OS), which was subsequently verified as being prognostic in the METABRIC validation cohort [3,4,5,9]. In addition, The Human Protein Atlas does not consider FGD3 expression as prognostic marker in breast cancer, whereas it reports that FGD3 has prognostic value in head and neck and cervical cancers. Concerning the setting of the cut-off for the analysis of FGD3 expression by IHC, we used the same criteria that were utilized in a previous study from our group that analyzed the expression of this gene in a series of young women with breast cancer [10]; a similar approach was used also by Willis et al. [9]. We found that lower FGD3 expression was significantly associated with higher incidence of recurrence and death from disease. Thus, we found that low expression of this gene was associated with a significant negative impact on both DFS and OS. This finding is in accordance with the inhibiting role of FGD3 on cell migration, that was purposed and demonstrated in other studies [4,5,9,10]. Interestingly, according to multivariate analysis, our results indicate that FGD3 expression represents an independent predictor of clinical outcome in breast cancer patients in terms of OS, second only to the AJCC stage, in agreement with the literature [9,14,26]. Similarly, in terms of DFS, FGD3 expression demonstrated to be an independent predictor of clinical outcome second only to the age at diagnosis. In fact, young age (<40 years) was the stronger predictor of recurrence, in accordance with literature [22].

A very interesting finding was that by stratifying patients by FGD3 expression and AJCC stage (stage I–II vs. stage III–IV), we identified patients with different outcome within the same AJCC stage subgroup. In particular, we highlighted that low FGD3 expression significantly worsen the prognosis of patients belonging to the same stage. In addition, it seems that FGD3 expression even exceeds the strength of AJCC stage in determining patients’ outcome; in fact, patients with advanced disease (stage III–IV AJCC) and high FGD3 expression did not significantly worse than patients with early-stage disease (stage I–II AJCC) but low FGD3 expression (Figure 4). These findings suggest that FGD3 expression as determined by IHC may add considerable prognostic information that outperforms that of all classical determinant of survival in breast cancer, except for the tumor stage. Further analysis in specific subgroups of patients for which a more precise prognostic assessment could be clinically useful in the choice of adjuvant treatment, such as those with lymph-node negative or lymph-node positive, Luminal A and Luminal B tumors, confirmed the role of FGD3 expression as evaluated by IHC to predict survival. These findings are in agreement with those of Willis et al., who reported the same results using the FGD3 mRNA expression [9]. Among the patients with triple-negative breast cancer, in the present study FGD3 expression confirmed to influence survival; however, the difference was not significant, possibly because of the small sample size. Hence, in the study of Willis et al., FGD3 mRNA expression was prognostic also in the subgroup of Basal tumors.

Previous studies [2,9] showed the potential inhibiting role of FGD3 on cellular migration and its influence in lymph node metastases. Thus, we analyzed the association between FGD3 expression and lymph-node involvement, and we found that lower FGD3 expression was significantly associated with the presence of at least one lymph node involved, in accordance with the literature. In addition, FGD3 staining intensity was significantly higher in patients without lymph node involvement compared to those with involved lymph nodes. In this series, however, the association between FGD3 staining intensity and involvement of more than 10 lymph nodes, that we found in a previous study limited to breast cancer in young women, was not confirmed [10]. A recent study of Willis et al. reported that estradiol stimulation is able to increase FGD3 mRNA expression level through the ESR1 binding site within the gene [9]. This finding may open interesting insights into the mechanisms by which FGD3 can influence breast cancer prognosis and at the same time may lead to formulate a hypothesis of FGD3 as a potential therapeutic target. Hence, the Collaborative Group on Hormonal Factors in Breast Cancer reported in two different studies that both oral contraceptive [27] and hormonal replacement therapy [28] increased the risk of breast cancer due to estrogen stimulation. On the other hand, these same studies observed that estrogen-induced breast cancers were less aggressive than the general population’s breast cancers. Therefore, we might now argue that FGD3 may have a role in this mechanism: estrogen stimulation could increase FGD3 expression, which in turn may determine a better prognosis in estrogen treated patients. Further studies are warranted to explore this hypothesis and its potential therapeutic implications.

## 4. Materials and Methods

### 4.1. Patients Selection and Data Collection 

We identified 401 women suffering from invasive breast cancer, surgically treated and followed between 1993 and 2019 at the Breast Unit of the Gynecology and Obstetrics Department, Careggi Hospital, University of Florence. We collected patients’ data from medical records, including surgical treatment of primary tumor and axillary lymph nodes, neoadjuvant and adjuvant treatment, clinical and pathological features of tumors, DFS and OS. After surgery, patients had follow-up visits every 6 months during the first 5 years and every year thereafter. Mammogram and an ultrasound scan of the breast was performed every 12 months during the follow-up period. The average follow-up interval was 70.8 months (range 1.2–276.5 months, considering that patients with a follow-up lasting less than 6 months were excluded from the study, except for patients who experienced recurrence or death during the follow-up, even if less than 6 months after surgery). All the patients gave their written informed consent to the use of tissue blocks for study purposes. 

### 4.2. FGD3 Expression

Immunohistochemical evaluation of FGD3 expression was performed on slides hours to days after deep sectioning of formalin-fixed, paraffin embedded tissue blocks. Slides were stored at 4 °C in order to test possible antigen recovery. In addition, antigen preservation was verified prior to FGD3 analysis by immunohistochemistry with internal positive anatomic controls. The use of this procedure prevented proteolytic degradation of the samples because it is known that formalin-fixed tissue within paraffin blocks maintain intact protein structures for even more than 20 years, in contrast with old slides in which proteolytic degradation may occur after some years. FGD3 protein expression was evaluated using a rabbit polyclonal antibody against FGD3 at a dilution of 1:750 (Sigma-Aldrich, St. Louis, MO, USA, Cat# HPA020963, RRID:AB 10609712). Paraffin removing, antigen recovery and antibody incubation were carried out using BenchMark ULTRA device (Ventana, Tucson, AZ, USA), according to a set protocol. The protein expression was evaluated using the detection system HRP ultra View Universal DAB Detection Kit (Ventana, Tucson, AZ, USA). Because lymphoid cells are known to always stain for FGD3, positive external control was obtained using tonsillar tissue, as well as using stromal lymphocytes as internal control. Negative control was obtained using a rabbit serum antibody (Normal, Dako Agilent, Carpinteria, CA, USA, Cat# A020602, RRID:AB_578507). The results were expressed as percentage of positive cells and as staining intensity (undetectable −, weak +, moderate ++, strong +++), as shown in Figure 1.

### 4.3. Cellular Reactivity Cut-Off Point

To evaluate the prognostic value of FGD3 expression, we compared the patients’ DFS and OS after dividing them into two groups according to cut-off points already used in a study conducted by our center published in 2019 [10], that subdivided the population in low-FGD3-expressing tumors (undetectable staining or weak staining with ≤30% positive cells) and high-FGD3-expressing tumors (all other cases: strong or moderate staining, or weak staining with >30% positive cells). This cut off was chosen in our former study by evaluating all possible cut-off (e.g., negative vs. any positive; negative and weak vs. moderate and strong, etc., and including all possible cut-off for percent of cells stained) and individuation of the strongest one as predictor of DFS and OS. In addition, this cut-off seems to be in line with that used by Willis et al., who also used the same categorization in four groups (undetectable −, weak +, moderate ++, strong +++) and in their study referred to “high expression” as a favorable prognostic feature [9]. 

### 4.4. Statistical Analysis

Data analysis was performed using IBM SPSS Statistics, version 25.0. The frequency distribution was assessed by Chi-square test. Disease-free interval and overall survival were calculated according to Kaplan–Meier method and evaluated by Log-rank test. Univariate Cox proportional hazards regression analysis was used to evaluate the effect of each prognostic factor on disease-free survival and overall survival. We used a multivariate Cox proportional hazards regression analysis, with forward selection of variables, to assess the independence of each prognostic variable. Data were analyzed and reported according to the Reporting Recommendations for Tumor Marker Prognostic Studies (REMARK) criteria [29].

## 5. Conclusions

The current study confirms that FGD3 expression is a significant and independent predictor of clinical outcome; in particular, high FGD3 expression was a protective factor against recurrence, death from disease and lymph node involvement. FGD3 expression allowed to distinguish within a group of patients with overlapping traditional prognostic assessment, those with a better clinical outcome.

Our study suggests the possible usefulness of introducing FGD3 testing in the clinical routine. Indeed, the use of a simple, cheap and widely accessible technique such as IHC for FGD3 evaluation may help to bring into the daily practice the progress of previous research studies carried out on very large cohorts of patients by using more sophisticated techniques. Our results must be interpreted with caution because of the retrospective nature of the study and the relatively small number of patients included. However, it is relevant that even in a single institution’s clinical case series, the expression of FGD3 allowed to identify patients with good or bad prognosis with a higher strength than most of the prognostic factors traditionally used in clinical practice. Indeed, the introduction of new strong prognostic factors such as FGD3 expression could help selecting patients at higher risk, for whom the choice of individualized treatment may result in improved outcome. Although large scale studies on this biomarker as prognostic factor in breast cancer already exist [3,4,5,9], further studies are warranted to confirm the role of IHC determination of FGD3 expression as prognostic factor in breast cancer patients.

## Figures and Tables

**Table 1:** Clinical and pathologic characteristics of patients: overall and according to FGD3 expression. Abbreviations: LVI lympho-vascular invasion; AJCC stage The American Joint Committee on Cancer staging of 2017; BCS breast conserving surgery; SLB sentinel lymph node biopsy; AD axillary lymph node dissection.

Characteristic	All	%	FGD3−	%	FGD3+	%	*p* Value
Age, median (range)	57 (22–89)	___	58.5 (23–87)	___	55.0 (22–89)	___	___
**Histological grade**							
G1	87	21.7	18	4.5	69	17.2	<0.001
G2	166	41.4	39	9.7	127	31.7	
G3	148	36.9	83	20.7	65	16.2	
**LVI**							
No	270	67.3	83	20.6	187	46.7	0.012
Yes	131	32.7	57	14.2	74	18.5	
**Molecular Subtype**							
Luminal A	137	34.2	27	6.8	110	27.4	<0.001
Luminal B	145	36.2	45	11.2	100	25.0	
HER2+	87	21.7	48	12.0	39	9.7	
Triple Negative	32	7.9	20	4.9	12	3.0	
**Ki67 expression**							
Ki67 ≤ 20%	185	46.1	40	10.0	145	36.1	<0.001 ^§^
Ki67 > 20%	208	51.9	96	24.0	112	27.9	
unknown	8	2.0	4	1.0	4	1.0	
**AJCC stage**							
I	227	56.6	56	14.0	171	42.6	<0.001
II	103	25.7	47	11.7	56	14.0	
III	66	16.5	34	8.5	32	8.0	
IV	5	1.2	3	0.7	2	0.5	
**Primary Tumor Surgery**							
BCS	281	70.1	89	22.2	192	47.9	0.037
Mastectomy	120	29.9	51	12.7	69	17.2	
**Axillary Lymph Node Surgery**							
SLB	268	66.8	72	18.0	196	48.8	<0.001
AD	133	33.2	68	17.0	65	16.2	
**Neoadjuvant Chemotherapy**							
No	352	87.8	122	30.4	230	57.4	0.775
Yes	49	12.2	18	4.5	31	7.7	
**Adjuvant Chemotherapy**							
No	250	62.3	70	17.5	170	44.8	<0.001
Yes	151	37.7	70	17.5	81	20.2	
**Hormonotherapy**							
No	115	28.7	52	13.0	63	15.7	0.006
Yes	286	71.3	88	21.9	198	49.4	
**Trastuzumab**							
No	345	86.0	109	27.2	236	58.8	0.001
Yes	56	14.0	31	7.7	25	6.3	
**Adjuvant Radiotherapy**							
No	99	24.7	37	9.2	62	15.5	0.554
Yes	302	75.3	103	25.7	199	49.6	

^§^ Contingency table performed on a total subtracted by the samples without any available data concerning Ki-67 index (*n* = 393).

**Table 2:** Multivariate Cox analysis for disease-free survival and overall survival. Abbreviations: Ref. Reference risk.

**Parameters**		**Disease-Free Survival**		
		**HR**	**95% CI**	***p***
Age at diagnosis	Age > 40 ys	Ref.		<0.001
	Age ≤ 40 ys	2.747	1.60–4.72	
FGD3 expression	High	Ref.		0.003
	Low	2.252	1.31–3.87	
AJCC stage	I, II	Ref.		0.012
	III, IV	2.018	1.16–3.50	
Hormonal receptor status	ER/PgR−	Ref.		0.033
	ER/PgR+	0.535	0.30–0.94	
**Parameters**		**Overall Survival**		
		**HR**	**95% CI**	***p***
AJCC stage	I, II	Ref.		<0.001
	III, IV	4.802	2.28–10.09	
FGD3 expression	High	Ref.		0.007
	Low	3.021	1.35–6.74	

**Table 3:** Association between FGD3 expression and lymph node involvement.

**FGD3 Expression**	**pN+**	**Total**	**%**	***p***
**Low** (− or ≤30% +)	64	140	45.7	<0.001
**High** (>30% +, ++, +++)	72	261	27.6	
**Total**	136	401	33.9	
**FGD3 Expression**	**pN+ ≥ 10**	**Total**	**%**	***p***
**Low** (− or ≤30% +)	14	64	21.9	0.882
**High** (>30% +, ++, +++)	15	72	20.8	
**Total**	29	136	21.3	

## Data Availability

The data presented in this study are available on request from the corresponding author.

## Supplementary Materials

The following are available online at https://www.mdpi.com/article/10.3390/cancers13153824/s1, Figure S1: DFS (a) and OS (b) according to FGD3 expression in patients initially treated by surgery, Figure S2: DFS (a) and OS (b) according to FGD3 expression in patients receiving neoadjuvant chemotherapy., Figure S3: DFS (a) and OS (b) according to FGD3 expression and lymph node status stratified groups., Figure S4: DFS (a) and OS (b) according to FGD3 expression in triple negative breast cancer patients.
