# Supplementary material for: Immunohistochemical Evaluation of FGD3 Expression: A New Strong Prognostic Factor in Invasive Breast Cancer
Source: Cancers (Basel). 2021 Jul 29;13(15):3824. doi: 10.3390/cancers13153824 (PMC8345064; doi:10.3390/cancers13153824)
Supplement: Supplementary file 1 [file cancers-13-03824-s001.zip › cancers-1277921-supplementary.pdf]

cancers

Supplementary Materials

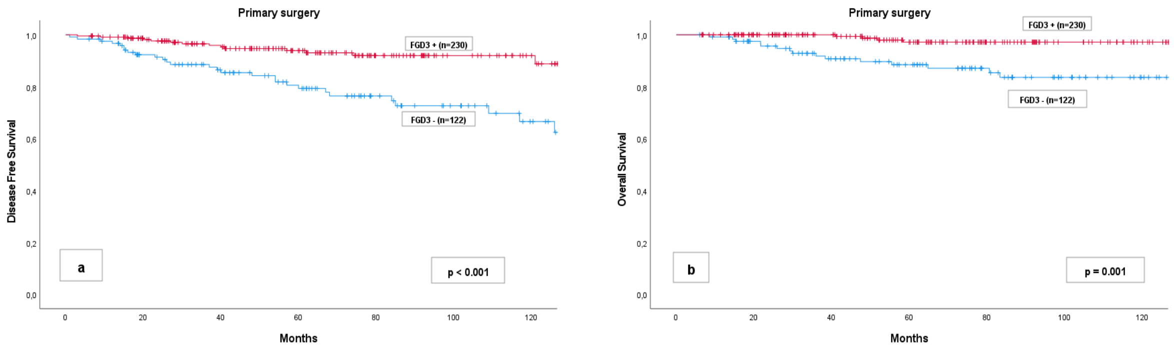

Figure S1. DFS (**a**) and OS (**b**) according to FGD3 expression in patients initially treated by surgery. Abbreviations: FGD3+: high FGD3 expression; FGD3-: low FGD3 expression.

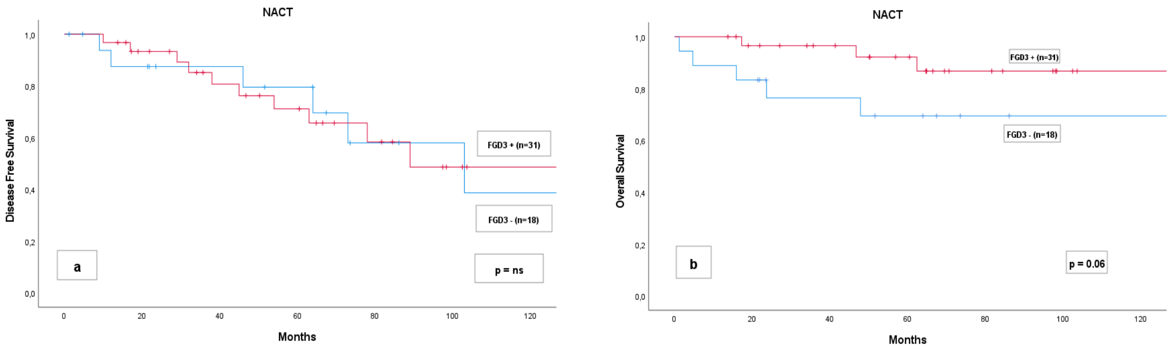

Figure S2. DFS (**a**) and OS (**b**) according to FGD3 expression in patients receiving neoadjuvant chemotherapy. Abbreviations: FGD3+: high FGD3 expression; FGD3-: low FGD3 expression.

cancers

MDPI

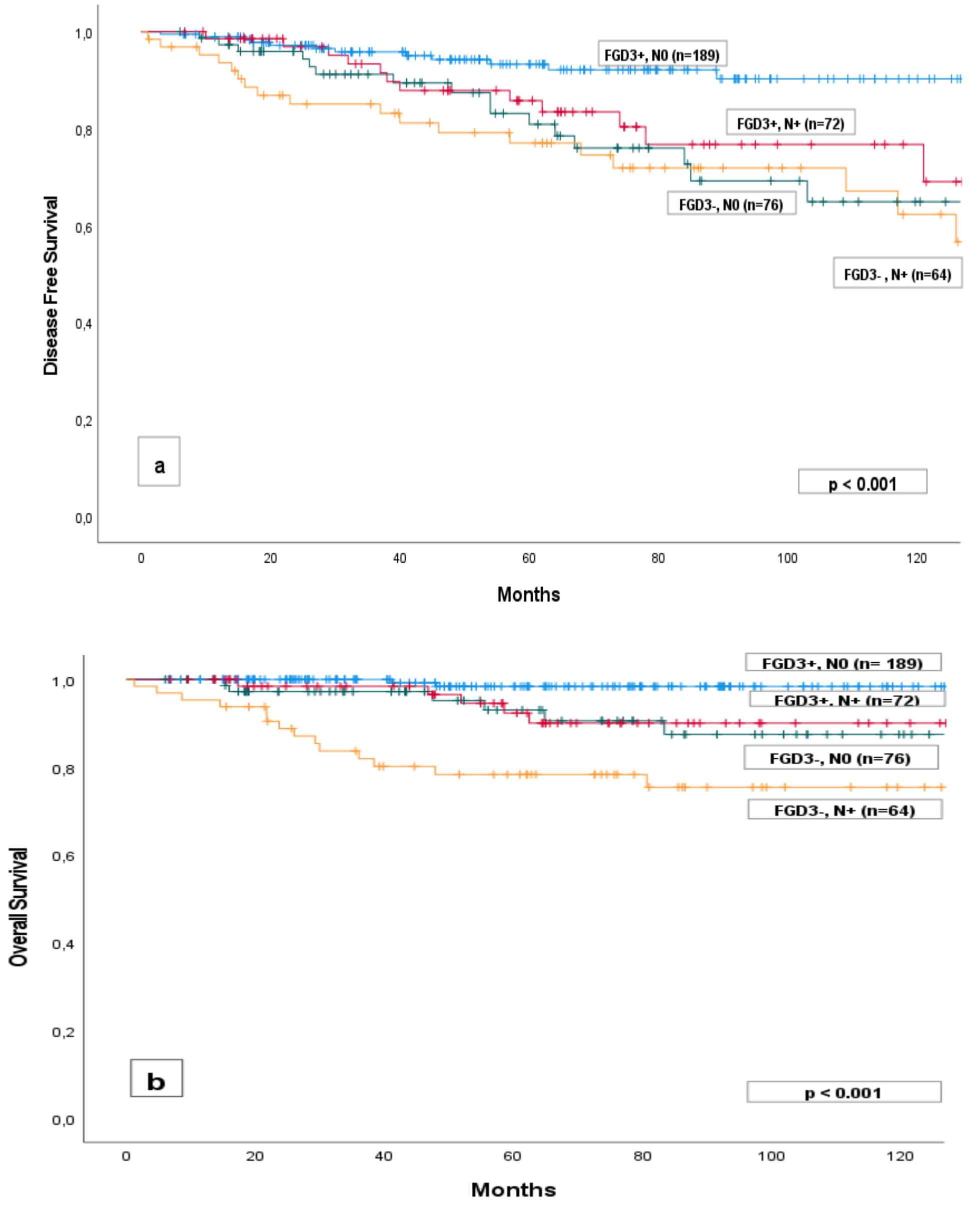

Figure S3. DFS (**a**) and OS (**b**) according to FGD3 expression and lymph node status stratified groups. Abbreviations: FGD3+: high FGD3 expression; FGD3-: low FGD3 expression; N0: lymph node negative patients; N+: lymph node positive patients.

a: FGD3+ N0 vs FGD3- N0: p<0.001; FGD3+ N0 vs FGD3+ N+: p=0.01; FGD3+ N0 vs FGD3- N+: p<0.001; FGD3- N0 vs FGD3+ N+: p= n.s.; FGD3- N0 vs FGD3- N+: p= n.s.; FGD3+ N+ vs FGD3- N+: p= n.s.;

b: FGD3+ N0 vs FGD3- N0: p=0.01; FGD3+ N0 vs FGD3+ N+: p=0.004; FGD3+ N0 vs FGD3- N+: p<0.001; FGD3- N0 vs FGD3+ N+: p= n.s.; FGD3- N0 vs FGD3- N+: p= 0.02.; FGD3+ N+ vs FGD3- N+: p= 0.03;

cancers

MDPI

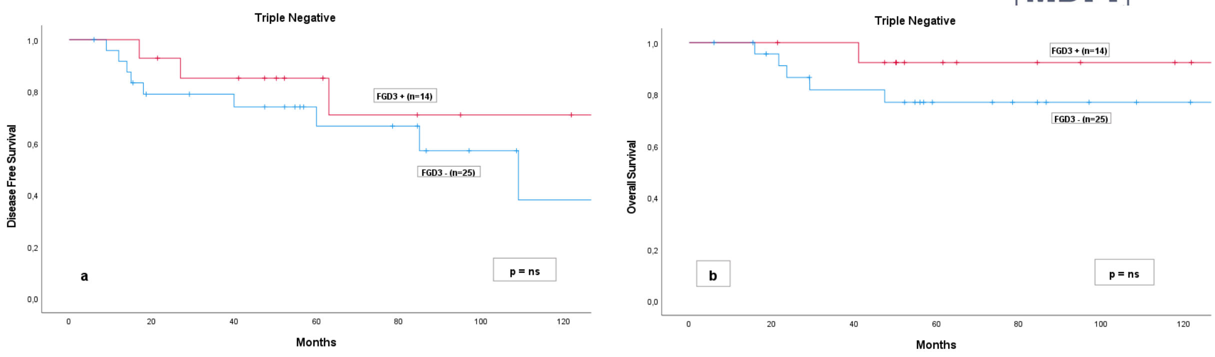

Figure S4. DFS (**a**) and OS (**b**) according to FGD3 expression in triple negative breast cancer patients. Abbreviations: FGD3+: high FGD3 expression; FGD3-: low FGD3 expression.
